# The LetA/S two-component system regulates transcriptomic changes that are essential for the culturability of *Legionella pneumophila* in water

**DOI:** 10.1038/s41598-018-24263-9

**Published:** 2018-04-30

**Authors:** Nilmini Mendis, Peter McBride, Joseph Saoud, Thangadurai Mani, Sebastien P. Faucher

**Affiliations:** Department of Natural Resource Sciences, McGill University, Sainte-Anne-de-Bellevue, Quebec, Canada

## Abstract

Surviving the nutrient-poor aquatic environment for extended periods of time is important for the transmission of various water-borne pathogens, including *Legionella pneumophila* (*Lp*). Previous work concluded that the stringent response and the sigma factor RpoS are essential for the survival of *Lp* in water. In the present study, we investigated the role of the LetA/S two-component signal transduction system in the successful survival of *Lp* in water. In addition to cell size reduction in the post-exponential phase, LetS also contributes to cell size reduction when *Lp* is exposed to water. Importantly, absence of the sensor kinase results in a significantly lower survival as measured by CFUs in water at various temperatures and an increased sensitivity to heat shock. According to the transcriptomic analysis, LetA/S orchestrates a general transcriptomic downshift of major metabolic pathways upon exposure to water leading to better culturability, and likely survival, suggesting a potential link with the stringent response. However, the expression of the LetA/S regulated small regulatory RNAs, RsmY and RsmZ, is not changed in a *relAspoT* mutant, which indicates that the stringent response and the LetA/S response are two distinct regulatory systems contributing to the survival of *Lp* in water.

## Introduction

*Legionella pneumophila* (*Lp*) is a bacterial contaminant of anthropogenic water distribution systems, where it replicates as an intracellular parasite of amoeba^1–3^. In the context of human infection, *Lp* preferentially targets alveolar macrophages causing a severe pneumonia termed Legionnaires’ disease^4^. The inhalation of *Lp* contaminated aerosols that are generated from water systems transmit the bacterium to the human lungs, where it proliferates^5–7^. Therefore, identifying the molecular mechanisms that *Lp* uses to survive in water is crucial, not only to better understand the operating system of the organism, but also for improving water systems management.

Transcriptional profiling revealed a drastic shut down of major pathways in *Lp* exposed to water^8^. Experiencing this starvation condition increases the resistance of *Lp* to antibiotics^8^, and likely other stresses. Indeed, one study exposed stationary phase *Lp* cultures, which are naturally more stress resistant than exponential (E) phase bacteria, to a nutrient-poor buffer^9^. The authors found that resistance to acid, hydrogren peroxide and antibiotic stresses acquired in the stationary phase were further enhanced by this treatment^9^. The current data suggests that *Lp* initiates unique transcriptomic and proteomic changes to adapt to and survive in water. At present, only a few genes or regulatory pathways are known to contribute to the survival of *Lp* in water^8,10–13^. The general silencing of gene expression in water seems to be orchestrated mainly by RpoS and the stringent response^14^.

Bacteria employ two-component systems (TCSs) to sense and respond to a variety of cues, ranging from temperature, antibiotics, quorum sensing autoinducer molecules and intermediates of the TCA cycle^15,16^. Upon sensing an activating environmental signal, the sensor kinase (SK) autophosphorylates a conserved histidine residue on its C-terminus. This phosphoryl group can then be shuttled to an aspartate residue on the cognate response regulator (RR), a DNA-binding protein, which will initiate the downstream transcriptional changes that allow the bacterium to adapt and respond to the aforementioned environmental stimulus^17,18^.

The LetA/S TCS of *Lp*^19^ is the ortholog of BarA/UvrY in *Escherichia coli*^20^ and GacS/GacA in *Pseudomonas spp*.^21^. LetS belongs to a family of tripartite sensor kinases which deviate from the traditional SK model. Similar to its well-studied counterpart BvgS in *Bordetella* spp., LetS architecture includes 3 major domains (transmitter (T), receiver (R) and histidine phosphotransfer (HPT) domains) that are involved in an internal phosphorelay activating its cognate response regulator LetA^22,23^. Upon activation, the T domain is phosphorylated by an ATP molecule. This, in turn, phosphorylates the R domain. Finally, the HPT domain receives the phosphate from R and relays it to the response regulator LetA^23^. The modular nature of this SK allows it to respond to multiple stimuli, where each stimulus leads to the activation of a different set of genes^23–25^. In *Lp*, LetS regulates a subset of post-exponential phase genes in response to nicotinic acid^23^.

The RRs orthologous to LetA activate transcription of the Csr/Rsm-type small regulatory RNAs (sRNAs)^23,26–30^. The CsrA/RsmA protein binds target mRNA and mainly serve to inhibit their translation^29–32^. Competitive binding of Csr/Rsm sRNAs to CsrA/RsmA relieves the inhibitory effects of the latter on its target mRNA, thereby promoting their translation^26,30–33^. Orthologs of LetA/S and their accompanying regulatory cascades are involved in the virulence phenotypes of a number of pathogens, including regulation of pathogenicity islands in *Salmonella*^34–36^, fimbriae and exopolysaccharide production in *E. coli*^37–39^, quorum sensing and production of extracellular lipase, cyanide and pyocyanin in *Pseudomonas* spp.^21,28,33,40–42^ and the ToxR virulence regulator in *Vibrio cholerae*^43^. Members of this TCS family are also involved in stress resistance, biofilm formation, the switch between glycolytic and gluconeogenic carbon sources, and iron acquisition in various bacterial species^30,33,37,39,44,45^. In *Lp*, LetA binds the promoters and positively affects transcription of three sRNAs, RsmX, RsmY and RsmZ^46,47^. RsmX is, however, absent in the *Lp* Philadelphia 1 strain and *L. longbeachae*^47^. CsrA in *Lp* represses post-exponential (PE) phase traits and promotes the expression of E phase genes^48^. RsmY/Z antagonizes CsrA and activates PE (in broth) and transmissive (*in vivo*) phase traits in *Lp*^19,46,48,49^. As a result, mutations within this cascade have been linked to attenuated virulence within host cells and reduced motility, as well as sensitivity to heat, oxidative and acid stress^19,46,48,50^.

The aim of the present study is to investigate the role of the LetA/S regulatory cascade in a relevant water model and to elucidate the transcriptome under LetS control in water. We report that LetS is responsible for a genome wide repression of metabolic pathways in response to water. Using Northern blotting, we confirm that LetS forms a regulatory cascade under the control of RpoS. Despite the dependence on RpoS we found that the cellular alarmone, ppGpp, is not the main activating signal for the sensor kinase, advocating for other environmental stimuli to be investigated.

## Results

### LetS is important for the culturability of *Lp* in water

**Figure 1:**
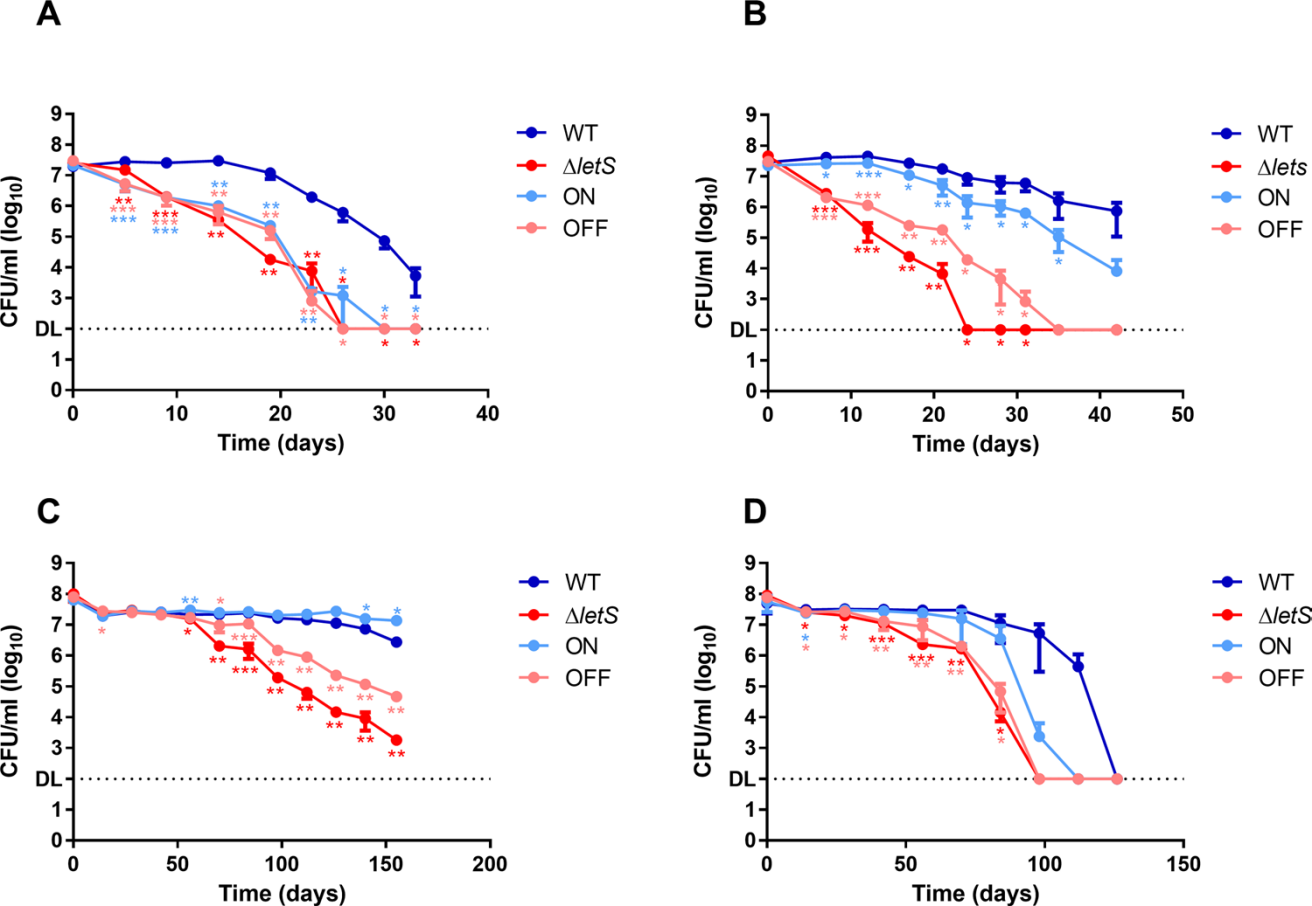
LetS increases the culturability of *Lp* in water. (**A** and **B**) CFU counts of the WT, ∆*letS* and the induced (ON) or uninduced (OFF) ∆*letS* + p*letS* was monitored in water at 42 °C. In panel A, ON was induced only in water, while in panel B, the ON strain was induced on agar prior to water exposure and during water exposure. Panel C and D show CFU counts of the WT, ∆*letS*, ON and OFF at 4 °C and 25 °C respectively. The ON strain in panels C and D was induced on agar prior to water exposure and during water exposure. Strains were suspended in water at an OD_600_ of 0.02. ON was induced with 0.1 mM IPTG. DL, detection limit. An unpaired, one-tailed Student’s t-test was used to assess statistical significance *versus* the WT. *p < 0.05; **p < 0.005; ***p < 0.0005.

The culturability of the ∆*letS* mutant was compared to that of the wild-type (WT) in water at 42 °C using CFU counts (Fig. 1A). An inducible plasmid carrying the *letS* gene (p*letS*) was introduced into ∆*letS* to complement the mutant strain. Expression of the plasmid-borne copy was induced with 0.1 mM IPTG, herein referred to as ON, or was left uninduced, herein referred to as OFF. A significant (*p* < 0.005) defect was observed in the ability of ∆*letS* to form colonies compared to the WT starting at early time points (Fig. 1A and B). Inducing the expression of *letS* in the ON strain solely in water did not correct this defect (Fig. 1A); however, when induced prior to and during water exposure, ON performed markedly better than the mutant (Fig. 1B). ∆*letS* and OFF behaved similarly to eachother. These results implicate the LetA/S two-component system as an important tool for the adaptation and potential survival of *Lp* in water at 42 °C.

Culturability of the strains was also tested at 4 °C and 25 °C to determine whether the defect observed at 42 °C was temperature specific. As a general trend, strains reached the detection limit slower as the incubation temperature decreased (Fig. 1), as previously described^51^. Similar to Fig. 1B, the loss of *letS* caused a defect when *Lp* was exposed to water at 4 °C and 25 °C, a phenotype that was complemented in the ON strain (Fig. 1C and D). At 25 °C, ON mirrored the WT strain better during the early stages of water exposure (Fig. 1D). IPTG is reported to be a stable inducer of gene expression with minimal decay in broth; however, its long-term stability in water over the course of several months has never been investigated to our knowledge. Alternatively, the uptake of IPTG may be compromised over the long-term. Indeed, *Lp* repressed the majority of its genes 24 hours after water exposure^8^, including channels and transporters that may be involved in IPTG uptake. Taken together, these experiments suggest that the LetA/S TCS is an important regulator that allows *Lp* to successfully adapt to and, likely, survive the nutrient poor aquatic environment.

The effect of the *letS* mutation on culturability of *Lp* was most pronounced at 42 °C (Fig. 1). Similarly, previous reports from our group found that the deleterious effect of some mutants was observed more readily at 42 °C than at lower temperatures^8,11,13^. A higher metabolic rate at elevated temperatures can explain the faster decline in CFU counts in mutant strains that are less fit than the wild-type. As such, when faced with starvation in water, internal energy sources would be depleted more rapidly at warmer temperatures. Accordingly, we expected the transcriptomic and physiological responses initiated by the LetA/S cascade to be apparent at earlier time points at 42 °C. Therefore, subsequent experiments were conducted at 42 °C to facilitate the study of the regulatory effects exerted by LetS.

### LetS influences morphological changes in water, pigment production and resistance to heat shock

**Figure 2:**
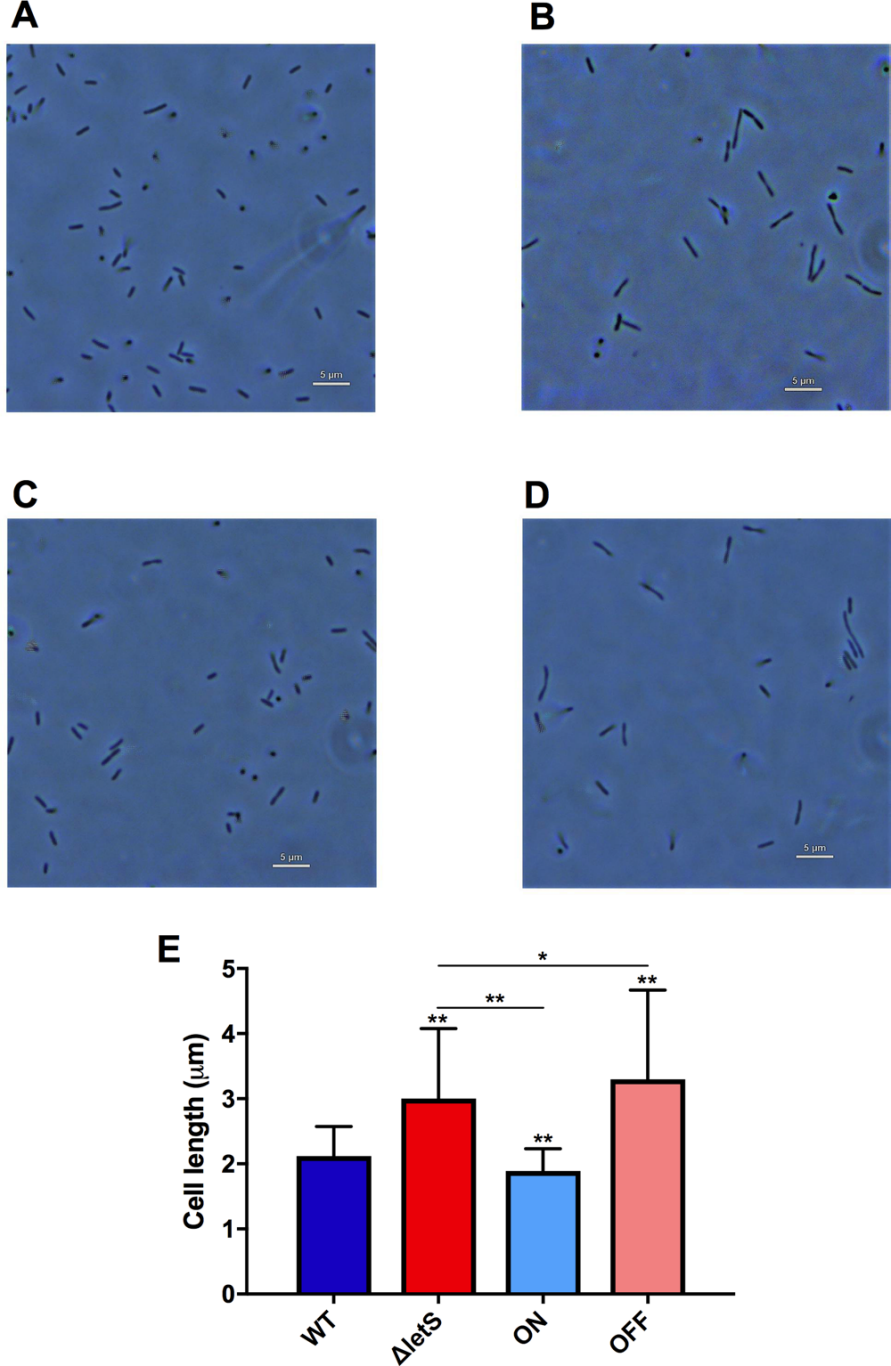
Deletion of *letS* affects the cell morphology of *Lp* in water. (**A**) Phase contrast microscopy was used to visualize morphological changes at 1000X magnification. A representative image of the WT (**A**), ∆*letS* (**B**), ON (**C**) and OFF (**D**) exposed to water for 24 hours are shown. In Panel E, Image J software was used to quantify the average length of 100 cells after exposure to water for 24 hours. The scale bar is equivalent to 5 μm. An unpaired, one-tailed Student’s t-test was used to assess statistical significance *versus* the WT, unless identified otherwise. *p < 0.05; **p < 0.0005.

WT, ∆*letS*, ON and OFF strains were exposed to water for 24 hours at 42 °C. Microscopic analysis determined that ∆*letS* cells were significantly (*p* < 0.0005) longer than WT cells after water exposure. Interestingly, the ON strain produced *Lp* cells that were significantly (*p* < 0.0005) shorter than the WT (Fig. 2A,C and E), which may be due to a higher level of *letS* expression. Notably, we observed that filamentous cells were more commonly found within the ∆*letS* and OFF populations than in the WT and ON populations. In accordance with previously published reports, cell size reduction was also observed in the post-exponential (PE) phase compared to the exponential (E) phase in the WT (Supplementary Figs S1 and S2)^49^. This reduction was absent in the *letS* and OFF strains. A strain over-expressing CsrA was similarly unable to reduce cell size upon entry into the PE phase^52^. Microscopic analysis highlights the contribution of LetS to cell size reduction in both the post-exponential phase and under the starvation condition of water, but not during the exponential phase of growth.

In agreement with previously published work^46,48^, ∆*letS* was defective for pigment production during the late PE phase (Supplementary Fig. S3). It is noteworthy that pigment production was not completely abolished in the ∆*letS* mutant which produced visible pigmentation. Nonetheless, the amount of pigment produced by the mutant was significantly (*p* < 0.0005) lower than the WT, a defect that was corrected in the ON strain (Supplementary Fig. S3).

**Figure 3:**
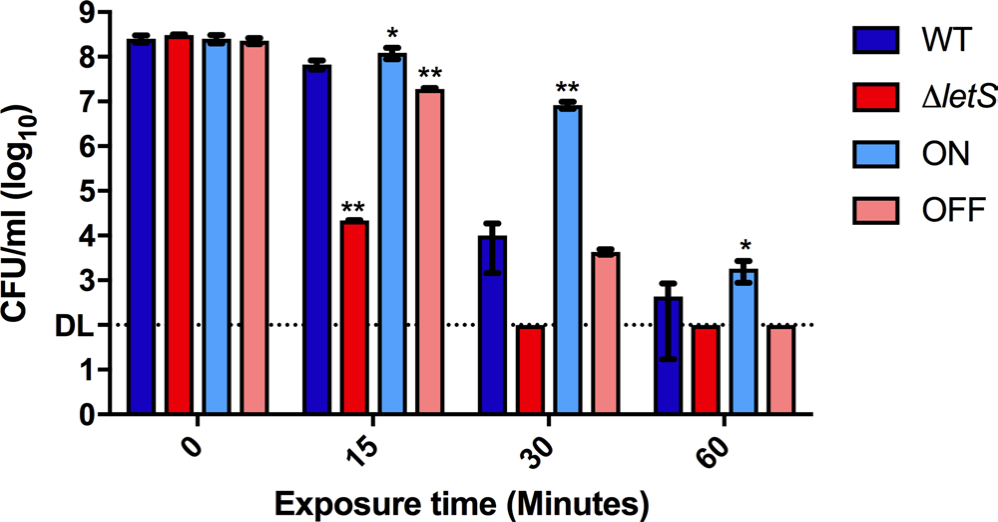
Deletion of *letS* affects sensitivity to heat shock. The WT, ∆l*etS*, ON and OFF strains were suspended in water for 2 hours and subsequently exposed to a 55 °C water bath for 15, 30 or 60 minutes. CFU counts were enumerated on CYE agar before and after the heat shock treatment. DL, detection limit. An unpaired, one-tailed Student’s t-test was used to assess statistical significance *versus* WT. *p < 0.05; **p < 0.005.

*Lp* frequently encounters high temperatures within man-made water distribution systems and is known to persist despite continuous heat treatments^53–56^. Therefore, we tested the ability of Δ*letS* to withstand heat shock in 55 °C water. WT CFU counts dropped by 5 orders of magnitude after 30 minutes at 55 °C, but remained above the detection limit after 60 minutes. In contrast, the *letS* mutant was more sensitive, as CFU counts dropped below the detection limit after a 30-minute heat shock treatment (Fig. 3). Expression of *letS* in ON afforded *Lp* an increased resistance to heat compared to the WT. The OFF strain resulted in lower heat tolerance compared to the WT and ON; however, its survival was markedly better than that of the *letS* mutant.

### Transcriptomic analysis of the LetS regulon in water

In order to identify genes affected by LetA/S in response to water, DNA microarrays were used to probe the transcriptomic differences between the WT, ∆*letS*, ON and OFF strains 2 hours after water exposure at 42 °C. Justification for using an early time point (2 h) were two-fold: 1) *Lp* is known to reduce transcription dramatically after 6 h in Fraquil^8^, and 2) we expect that the regulatory changes initiated by the LetA/S TCS are important for early adaptation in *Lp* as evidenced by the need for *letS* induction on agar prior to water exposure to achieve proper complementation (Fig. 1). To extract sufficient RNA for transcriptomic analysis, a high cell density was required. Therefore, the survival defect observed at low cell density (Fig. 1 – OD_600_ 0.02) was confirmed at high cell density (Supplementary Fig. S4 - OD_600_ 1).

**Figure 4:**
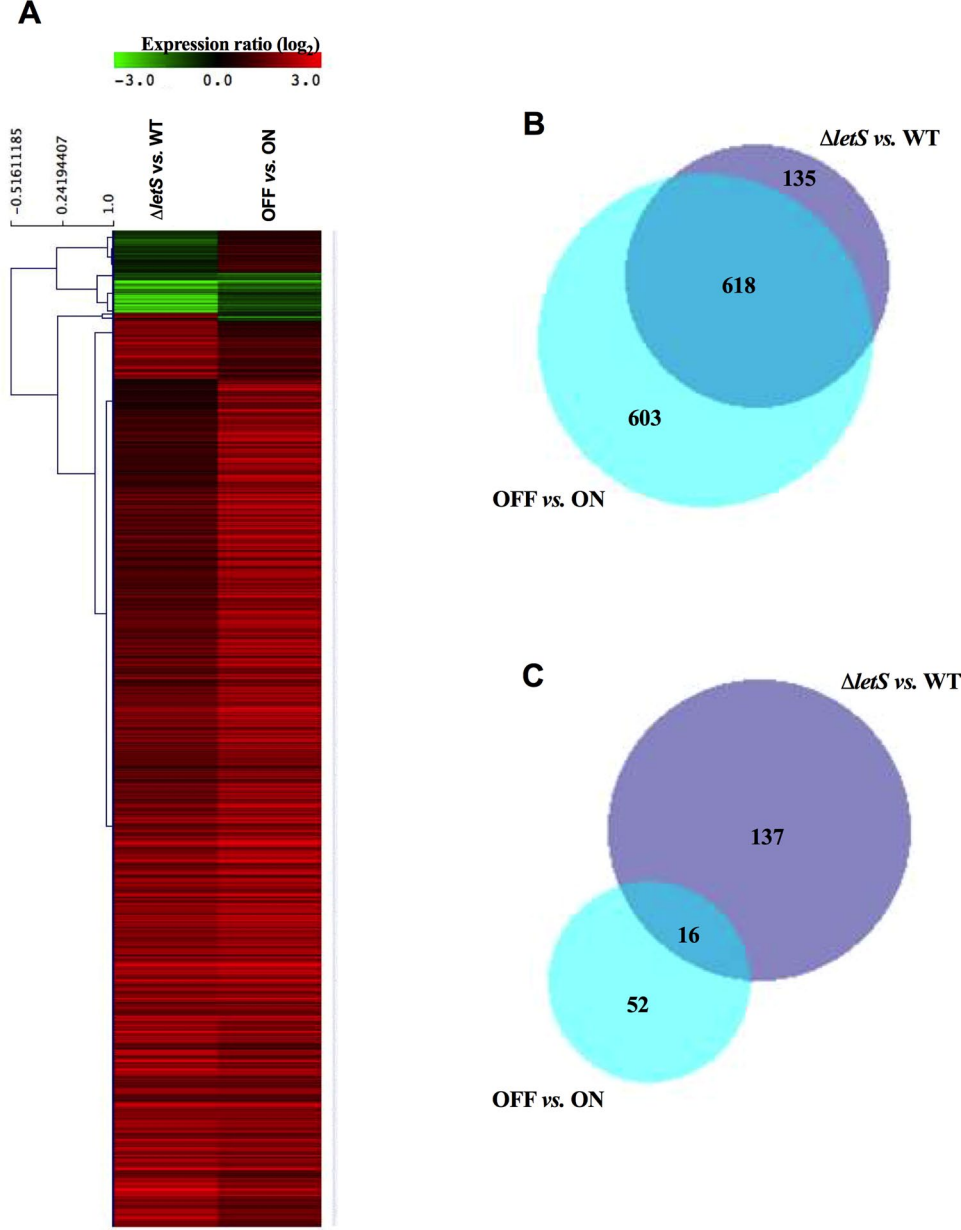
The absence of *letS* leads to ectopic up-regulation of gene expression in water. (**A**) A heat map showing genes differentially expressed in ∆*letS* compared to the WT (left), and in OFF compared to ON (right) (ratio to control value of ± 2-fold with a *p* < 0.05). Genes that are up-regulated in ∆*letS* and OFF are shown in red; genes that are down-regulated are shown in green. The number of up- (**B**) or down-regulated (**C**) genes that are shared between the ∆*letS vs*. WT and OFF *vs*. ON groups are shown in Venn diagrams.

**Figure 5:**
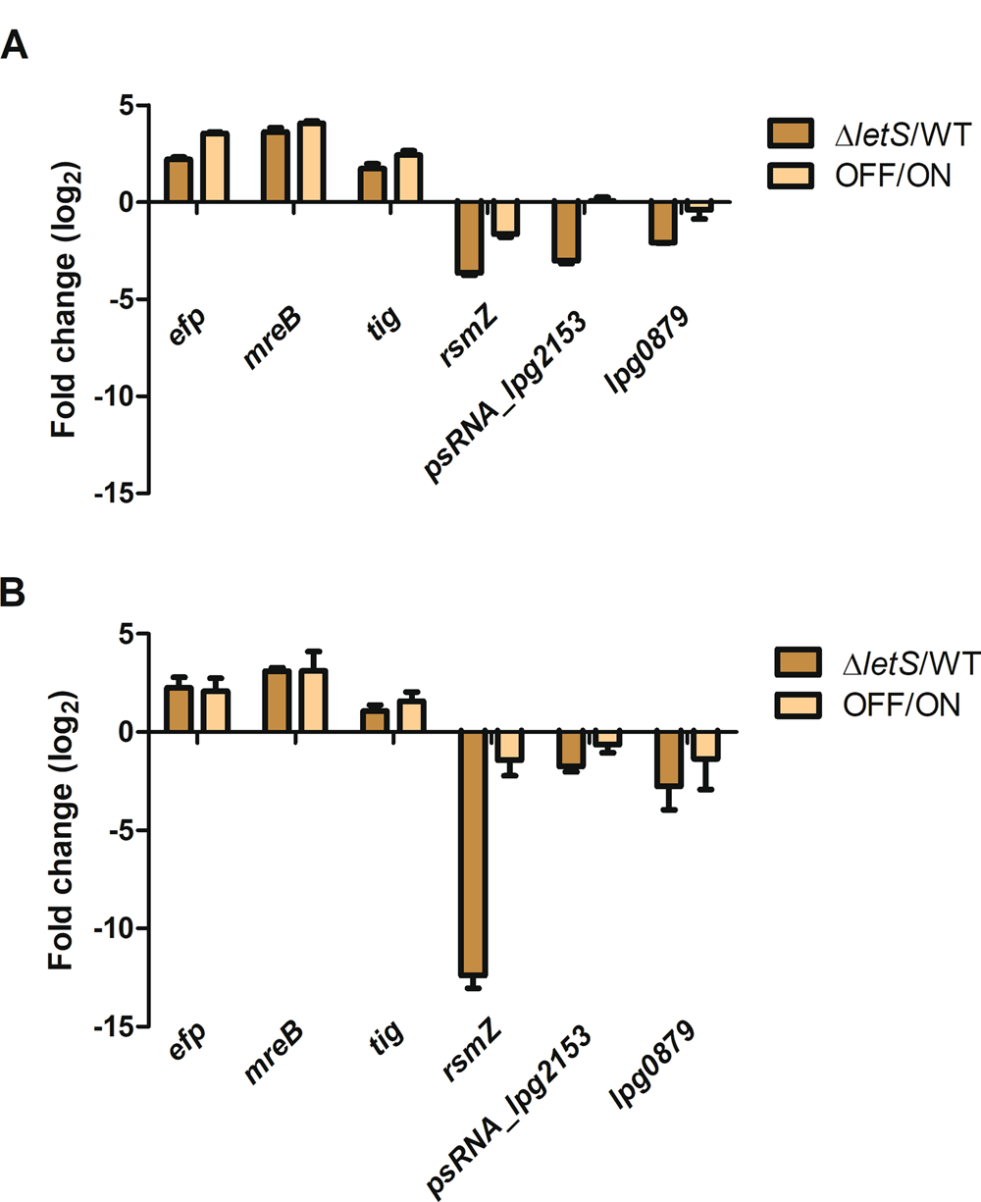
qPCR validates the DNA microarray analysis of the LetS regulon. RT-qPCR (**A**) was used to analyze the expression pattern of three up-regulated and three down-regulated genes in the transcriptomic analysis of WT *vs*. Δ*letS* (**B**). The fold change of each gene in the *letS* mutant (*vs*. the WT) and the OFF strain (*vs*. ON) are presented.

Gene expression profile of ∆*letS vs*. the WT was compared to that of OFF *vs*. ON in Fig. 4. The absence of *letS* led to the up-regulation of a large portion of genes (Fig. 4). Among the significantly (*p* < 0.05) up-regulated open reading frames (ORFs), 569 genes and 49 annotated sRNAs (a total of 618 ORFs) were shared in both ∆*letS* and OFF compared to the WT and ON respectively (Fig. 4B, Supplementary Table S1). On the other hand, 16 genes, including the two sRNAs RsmY and RsmZ, were significantly (*p* < 0.05) down-regulated in both the *letS* mutant and OFF (Fig. 4C, Supplementary Table S1). qPCR was performed on six randomly chosen genes; three were upregulated and three were downregulated in Δ*letS* compared to the WT (Fig. 5). The fold change pattern observed in the microarray analysis was mirrored in the qPCR results (Fig. 5).

**Figure 6:**
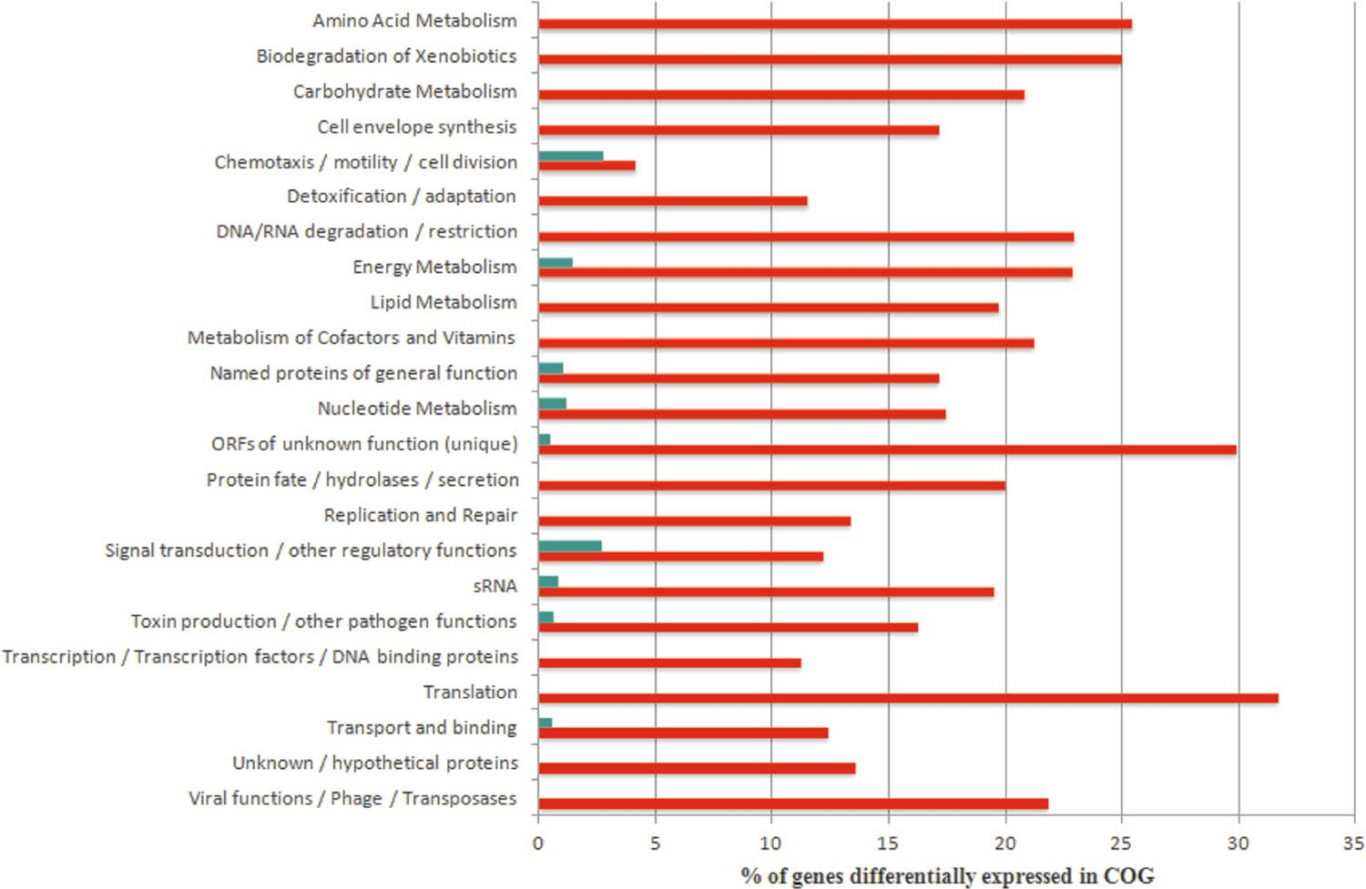
Clusters of orthologous groups (COGs) analysis of genes affected by the absence of *letS* in water. Data represents the differentially expressed genes that are common to both the ∆*letS vs*. WT group and the OFF *vs*. ON group. Red bars indicate the percentage of genes upregulated in each COG, while green bars indicate the percentage of genes that are downregulated in each COG category.

Genes that are differentially and significantly (*p* < 0.05) expressed in both ∆*letS* and OFF, relative to the WT and ON respectively, were categorized into clusters of orthologous groups (COGs) (Fig. 6). The cellular functions most affected by LetS were those associated with exponential growth or nutrient rich conditions and were mostly negatively affected by LetS in water (Fig. 6). These include “Translation”, “Amino acid metabolism”, “Lipid metabolism” and “Energy metabolism”. Notably, 30 of the 38 “Translation” genes that are normally down-regulated by LetS in water encode 30 S or 50 S ribosomal proteins (Fig. 6, Supplementary Table S1). It is also noteworthy that the *rpoD* transcript coding the housekeeping sigma factor was also highly up-regulated in water in the absence of *letS* (Table 1).

**Table 1:** Select genes differentially regulated in ∆*letS vs*. WT and OFF *vs*. ON.

Lpg #	Gene Product	Gene	log_2_ (*letS/WT*)*	log_2_ (OFF/ON)*
**Upregulated**				
**Amino Acid Metabolism**				
Lpg0932	shikimate kinase		1.05	1.48
Lpg1610	glutamate-5-kinase (gamma-glutamyl kinase)	proB	1.72	1.33
Lpg2278	4-hydroxyphenylpyruvate dioxygenase (legiolysin) oxidoreductase protein (hemolysin)	hpd	2.12	1.45
Lpg0890	cystathionine beta-lyase (cystathionine gamma lyase)	metC	1.53	1.40
Lpg2951	cystathionine beta synthase (cysteine synthase)		1.62	1.98
Lpg0725	serine hydroxymethyltransferase	glyA3	2.60	2.80
**Carbohydrate Metabolism**				
Lpg2887	phosphomannose isomerase GDP mannose pyrophosphorylase (mannose-1-phosphate guanylyltransferase/mannose-6-phosphate isomerase)	rfbA	1.57	2.14
Lpg0939	2-dehydro-3-deoxy-phosphogluconate aldolase	eda	1.82	1.71
Lpg0417	6-phosphogluconolactonase	pgl	1.91	2.46
Lpg0805	phosphoenolpyruvate synthase		2.32	2.85
Lpg2352	malate dehydrogenase	mdh	1.39	2.28
Lpg2792	triosephosphate isomerase (TIM)	tpiA	2.72	2.66
Lpg0138	glyceraldehyde 3-phosphate dehydrogenase	gap	1.44	2.40
**Cell Envelope Synthesis**				
Lpg1753	UDP-N-acetylmuramate:L-alanyl-gamma-D-glutamyl-meso-diaminopimelate ligase (murein peptide ligase)	mpl	1.83	2.15
Lpg0840	polysialic acid capsule expression protein (carbohydrate isomerase) (KpsF/GutQ family protein)		1.45	2.08
Lpg2544	membrane-bound lytic murein transglycosylase A	mltA	1.12	1.87
Lpg0748	LPS biosynthesis protein (pseudaminic acid biosynthesis and flagellin acetamidinic modification?)		2.10	2.35
Lpg0811	rod shape determining protein MreB (regulator of FtsI)	mreB	3.07	2.41
**Motility & Cell Division**				
Lpg2891	sporulation initiation inhibitor protein Soj, chromosome partitioning protein ParA	soj	1.38	1.45
Lpg1553	septum site determining protein MinC (FtsZ assembly inhibitor)	minC	1.05	1.86
Lpg1724	septum site-determining protein MinD (cell division inhibitor (membrane ATPase) activates MinC)	minD	1.24	1.86
**Detoxification/Adaptation**				
Lpg0047	chloramphenicol acetyltransferase (highly similar to antibiotic acetyltransferase)		1.54	1.22
Lpg0426	cold shock protein CspH	cspD	1.04	1.71
Lpg1060	cold shock domain family protein, COG1278: cold shock proteins		1.02	2.62
Lpg1971	organic hydroperoxide resistance protein, COG1764:predicted redox protein, regulator of sulfide bond formation		1.81	2.97
Lpg2967	superoxide dismutase	sodB	1.82	1.72
Lpg1861	ATP-dependent Clp protease, proteolytic subunit ClpP	clpP	1.41	2.15
Lpg1423	TPR domain protein (heat shock protein) N-acetylglucosaminyl transferase		1.93	1.58
**DNA/RNA Degradation**				
Lpg1373	ribonuclease HII	rnhB	1.37	1.11
Lpg1383	ribonuclease HI	rnhA	1.00	1.73
Lpg1869	ribonuclease III (dsRNA-specific ribonuclease) (RNAse III, dsRNA)	rnc	2.04	2.24
Lpg0609	alanyl tRNA synthetase	alaS	1.16	2.57
Lpg2004	S-adenosylmethionine:tRNA ribosyltransferase-isomerase	queA	1.96	2.09
Lpg2012	ribonuclease PH (RNAse PH)	rph	2.33	2.62
**Energy Metabolism**				
Lpg2981	ATP synthase epsilon chain, ATP synthase F1 epsilon subunit	atpC	2.25	2.74
Lpg2982	H + -transporting two-sector ATPase, ATP synthase F1 subunit beta	atpD	1.62	2.12
Lpg2986	ATP synthase F0, B subunit	atpF	1.12	1.75
Lpg2779	NADH dehydrogenase I, K subunit (NADH-ubiquinone oxidoreductase, chain K)	nuoK	1.56	1.32
Lpg2787	NADH dehydrogenase I, C subunit (NADH-ubiquinone oxidoreductase, chain C)	nuoC	1.99	2.18
**Icm/Dot Genes Effectors**				
Lpg0483	LegA12	legA12	2.25	2.07
Lpg2283	small ORF (132aa)	celLp6	1.43	2.42
Lpg0621	sidA	sidA	1.44	1.01
Lpg0963	ORF		2.25	1.94
Lpg1110	ORF	lem5	2.12	2.65
Lpg2298	inclusion membrane protein A	legC7/ylfA	1.52	2.07
Lpg2793	LepA, interaptin	lepA	1.24	2.27
Lpg2999	CG6763 gene product (eukaryotic homologs?)	legP	1.22	2.14
**Lipid Metabolism**				
Lpg0102	3-oxoacyl-[acyl carrier protein] synthase (beta-ketoacyl synthase)	fabF	1.84	1.30
Lpg1395	3-oxoacyl-(acyl carrier protein) reductase	fabG	2.07	1.44
Lpg1854	enoyl reductase (NADH dependent enoyl ACP reductase) (enoyl [acyl carrier protein] reductase (NADH2))	fabI	1.26	1.92
Lpg2228	3-oxoacyl (acyl carrier protein) synthase III		1.57	2.03
Lpg0729	phosphatidylglycerophosphatase A (PgpA)	pgpA	1.73	2.54
Lpg0920	phosphatidylglycerophosphatase B (Pap2)		1.34	2.90
Lpg1414	glycerol kinase (probable carbohydrate kinase)		1.88	1.48
**Nucleotide Metabolism**				
Lpg0218	phosphoribosylaminoimidazole carboxylase, catalytic subunit PurE	purE	1.92	1.69
Lpg1181	CTP synthase PyrG	pyrG	1.52	2.99
Lpg1411	adenylate kinase (ATP-AMP transphosphorylase)	adK	1.67	2.06
Lpg1676	phosphoribosylformylglycinamidine synthase I (FGAM synthase I)	purQ	1.21	1.60
Lpg1678	phosphoribosylformylglycinamidine synthase II (FGAM synthase II)	purL2	1.33	2.10
**Protein Fate & Secretion**				
Lpg0316	preprotein translocase, SecE subunit	secE	1.12	1.28
Lpg1362	type II protein secretion LspG (general secretion pathway protein G)	gspG	1.60	1.81
Lpg1463	preprotein translocase; secretion protein SecA	secA	2.70	2.49
Lpg1871	signal peptidase I (lepB-1)	lepB-1	1.62	1.56
Lpg2002	transmembrane protein YajC, preprotein translocase subunit	yajC	1.64	1.62
Lpg2791	preprotein translocase, SecG subunit	secG	2.30	2.94
**Replication & Repair**				
Lpg0356	single strand binding protein	ssb	2.09	2.07
Lpg0691	DNA topoisomerase IV subunit B (DNA gyrase subunit B)	parE	1.49	2.76
Lpg1417	DNA gyrase, A subunit	gyrA	2.31	2.35
Lpg1576	Holliday junction DNA helicase RuvB	ruvB	1.47	1.96
Lpg1801	RecA bacterial DNA recombination protein (recombinase A)	recA	1.74	1.68
**Virulence Related Genes**				
Lpg0704	enhanced entry protein EnhA	enhA	1.29	1.25
Lpg0791	macrophage infectivity potentiator (mip)	mip	1.27	1.83
Lpg2564	LvrA		1.69	1.27
Lpg0447	LphA (DotK) (OmpA family protein)	lphA	1.80	2.51
Lpg0448	IcmM (DotJ)	icmM	1.37	1.75
Lpg0450	IcmK (DotH) (TraN)	icmK	1.13	1.24
Lpg2674	DotD (TraH)	dotD	1.08	2.12
Lpg1862	trigger factor TF (FKBP-type peptidyl prolyl cis-trans isomerase)	tig	2.62	1.64
Lpg2702	stringent starvation protein A (transcription activator)	sspA	1.42	1.77
**Transcription**				
Lpg2624	transcription elongation factor GreA	greA	1.66	3.10
Lpg2934	transcription termination factor rho		1.52	1.68
Lpg0232	transcriptional regulator np20 (Fur family) (ferric uptake)	np20	2.26	2.39
Lpg0542	DNA binding protein Fis (recombinational enhancer binding protein; factor-for-inversion stimulation protein)	fis	2.15	2.63
Lpg1743	Fis transcriptional activator (factor for inversion stimulation) (DNA-binding protein)	fis	1.05	1.00
Lpg2361	RNA polymerase sigma 70 factor (sigma factor RpoD)	rpoD	1.45	2.00
**Translation**				
Lpg0287	translation elongation factor P (EF-P)	efp	1.28	1.60
Lpg0339	50S ribosomal protein L14	rplN	1.05	1.58
Lpg0341	50S ribosomal protein L5		1.18	1.74
Lpg1592	30S ribosomal protein S6	rpsF	3.92	3.25
Lpg1711	ribosome recycling factor (ribosome releasing factor)	frr	1.34	2.09
Lpg1713	translation elongation factor Ts (EF-Ts) (ubiquitin associated domain:elongation factor Ts)	tsf	2.51	2.27
Lpg1714	30S ribosomal protein S2	rpsB	1.62	1.39
Lpg2713	translational initiation factor IF-3	infC	2.80	2.65
**Transport & Binding**				
Lpg1277	ABC transporter ATP binding protein (abcT3) (multidrug resistance ABC transporter) (hemolysin secreting ATP binding protein)	abcT3	2.80	2.64
Lpg2245	C4-dicarboxylate transport protein (Na+/H+dicarboxylate symporter)	dctA	1.51	1.66
Lpg2321	serine transporter	sdaC	1.79	2.75
Lpg2475	hydrogenase expression/formation protein (hydrogenase nickel incorporation protein HypB)	hypB	1.81	1.59
Lpg2476	hydrogenase nickel incorporation protein HypA	hypA	2.71	2.88
Lpg2658	ferrous iron transporter A	feoA	1.89	1.79
Lpg2878	cobalt/magnesium uptake transporter	corA	1.39	2.30
**Small Regulatory RNA**				
	Small regulatory RNA	lprC	1.08	2.02
	Small regulatory RNA	lpr0035	1.65	1.91
	Small regulatory RNA	lprD	1.67	2.26
**Downregulated**				
	Small regulatory RNA	rsmZ	−14.43	−1.32
	Small regulatory RNA	rsmY	−3.69	−4.21
Lpg1337	flagellar protein FliS	fliS	−2.77	−1.72
Lpg1170	pyruvate formate lyase-activating enzyme PflA		−2.32	−1.38
Lpg0605	nitrogen fixation protein (Fe-S cluster formation) NifU		−2.28	−1.36
Lpg0894	cytokinin oxidase (cytokinin dehydrogenase)		−2.92	−1.77
Lpg2829	SidH (myosin-like protein) Icm/Dot Effector	sidH	−2.81	−1.04
Lpg1169	hypothetical (dioxygenase)		−1.04	−1.05
Lpg1080	deoxyguanosine triphosphate triphosphohydrolase (dGTP triphosphohydrolase)		−2.70	−1.10
Lpg1925	ORF of Uknown Function		−3.50	−1.92
Lpg0995	ORF of Uknown Function		−1.36	−2.20
Lpg2458	sensory box histidine kinase (two-component sensor histidine kinase, signal transducing histidine kinase)		−3.33	−1.15
Lpg0879	two component response regulator with GGDEF domain (regulatory components of sensory transduction system)		−2.92	−1.49
Lpg0627	type IV pilin (competence and adherence associated pilin PilA)	pilE3	−2.86	−1.43
Lpg0628	type IV fimbrial biogenesis PilY1-related protein		−2.26	−1.09
Lpg1949	Icm/Dot Effector	lem17	−1.34	−1.31

^*^Only significant values (P < 0.05) are shown.

### The LetA/S cascade is linked to the RpoS regulatory cascade, but its activation is ppGpp-independent

**Figure 7:**
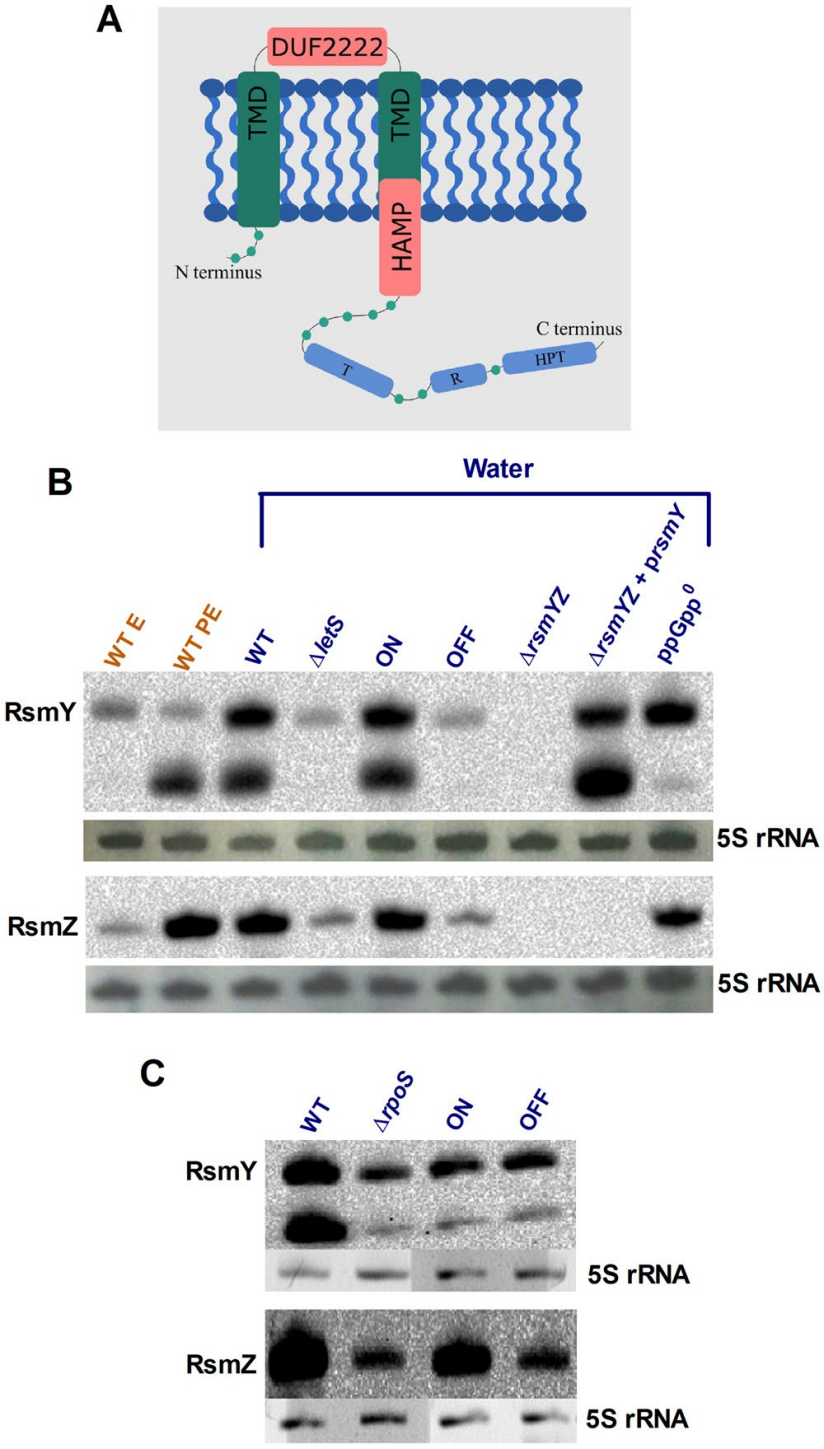
LetS topology and the impact of the stringent response elements, RpoS and ppGpp, on RsmY/Z expression. (**A**) Topology of the LetS protein was determined using the NCBI CDD Web server, as well as TMHMM v.2.0 and TOPCON software. Transmembrane domains (TMD) are represented by green boxes. Two putative signal sensing domains (pink boxes) are also predicted; a DUF2222 domain is located between the two TMD, and a HAMP domain overlaps the C-terminus of the second TMD. The transmitter (T), receiver (R) and phosphotransfer (HPT) domains that are involved in signal transduction are represented by light blue boxes. (**B**) The sRNAs RsmY (top) and RsmZ (bottom) under LetS control were probed to determine their levels in water and the influence of ppGpp on their expression. The first two lanes represent the WT strain grown in rich broth to the exponential (E) or the post-exponential (PE) phase. The remaining wells represent the respective strains exposed to water for 2 hours. (**C**) Impact of RpoS on the expression of RsmY/Z. The sRNAs RsmY (top) and RsmZ (bottom) under LetS control were probed to determine their levels in water. The WT (JR32), *rpoS* mutant, the induced (ON) or uninduced (OFF) ∆*rpoS* + p*rpoS* strains were exposed to water for 2 hours. 1 μg of RNA was loaded into each well. Acrylamide gels were stained with ethidium bromide to visualize the 5 S rRNA loading control (shown beneath the respective blots). See Supplementary Figs S5 and S6 for complete gel and blot images.

*Lp* responds to water by shutting down major metabolic gene groups, such as replication, transcription, translation and amino acid metabolism^8^. In the absence of *letS*, *Lp* is unable to mount such a response, which likely results in overconsumption of internal resources. The transcriptomic response of ∆*letS* shows similarity to the response of an *rpoS* insertion mutant exposed to water; both mutants demonstrate abhorrent up-regulation of gene transcription in response to water^14^. Moreover, there is considerable phenotypic overlap between the activation of the stringent response and the LetA/S cascade both *in vitro*^19,57,58^ and *in vivo*^19,59^. As a result, LetS has been proposed to respond to the stringent response alarmone, ppGpp, during starvation^60^. We decided to investigate whether this hypothesis holds true when *Lp* is exposed to water. The NCBI Conserved Domain Database (CDD) was used to identify protein domains within LetS that could be responsible for environmental signal recognition. Figure 7A is a graphical representation of LetS topology as predicted by the bioinformatic tools described herein. The transmitter (T), receiver (R) and histidine phosphotransfer (HPT) domains, characteristic of tripartite sensor kinases, are depicted by blue boxes, preceded by two transmembrane domains (TMDs) in green. In addition to these domains that comply with a previous study^23^, we report the presence of signal sensing domains and their topology, thereby fine-tuning our knowledge of LetS structure. The N-terminus of LetS contains two conserved signal sensing domains; DUF2222 and HAMP. The former is located between residues 37–180, while the latter spans residues 185–241. The two TMDs are located between residues 15 to 34 and 182 to 204 (TMHMM), or 11 to 31 and 181 to 201 (TOPCONS). While the DUF2222 domain is found between the two helices, the N-terminus of the HAMP domain overlaps the second transmembrane (TM) helix by 19 (TMHMM) or 16 residues (TOPCONS) (Fig. 7A). Therefore, approximately 30% the HAMP domain lies within the membrane, and 70% in the cytoplasm. LetS may be activated by an extracellular (via DUF2222), intracellular (via HAMP) or by an intermembrane (via the TM region of HAMP) signal. Here, we investigated the possibility that ppGpp may act as an intracellular signal that activates the LetA/S cascade in response to water exposure as previously postulated^48,61^.

The small RNAs (sRNAs) RsmY and RsmZ were used as reporters for the activation of LetS using Northern blotting. A ∆*relAspoT* strain (ppGpp°), incapable of producing ppGpp^14^, was used to determine the effect of ppGpp on RsmY/Z production. The probe for RsmY detected two bands (Fig. 7B). Similar banding patterns are reported for RsmZ in *Pseudomonas fluorescens* and RsmY in *P. aeruginosa*^62–64^. The top band (~110 nucleotides) was considered the active sRNA as per previous reports^46,65^. Corroborating previous studies^65,66^, the levels of RsmZ increase when cells enter the PE phase (Fig. 7B – bottom panel). This increase was, however, absent in RsmY (Fig. 6 – top panel). We postulate that the increase in RsmY may occur later during the PE phase. Nevertheless, both sRNAs were strongly expressed in water. ∆*letS* and OFF expressed both RsmY/Z at basal levels, while ON recovered WT level expression of RsmY/Z in water. The basal levels of RsmY/Z observed in the *letS* mutant is likely due to strong promoters preceding the sRNA coding regions, as the E phase WT cells exhibited similar bands (Fig. 7B). The expression of RsmY/Z was abolished in ∆*rsmYZ*; RsmY expression was recovered to WT levels when *rsmY* is expressed *in trans*. The absence of the alternative sigma factor, RpoS also resulted in a significant decrease in RsmY/Z expression (Fig. 7C), corroborating previous findings that the LetA/S cascade is RpoS-dependent^65^. Importantly, the ppGpp null mutant had WT levels of the RsmY transcript and a slightly lower level of RsmZ compared to the WT, suggesting that the alarmone is dispensable for LetS activation in water and expression of the effector sRNAs.

## Discussion

*Legionella pneumophila* (*Lp*) is a resident of natural and man-made water systems, and uses aerosols generated by the latter to infect human hosts^5^. *Lp* leads a biphasic lifestyle, alternating between the replicative phase and transmissive phase during infection, and between the exponential (E) and post-exponential (PE) phase in broth culture^58,67^. The former stages are characterized by nutrient abundance. In contrast, the hallmark of the transmissive/PE phase is nutrient deprivation causing morphological changes, a transcriptomic shift, stress resistance and virulence phenotypes^58,61,68^. The LetA/S two-component system is a key tool for differentiation of *Lp* in response to starvation, both in artificial medium and inside the host^46,50,66,69,70^. The established role of LetA/S in adaptation to low nutrient conditions led us to test its contribution to surviving starvation experienced in water.

The absence of *letS* did not affect the growth rate of the mutant on solid medium. Pyomelanin production, which is linked to iron acquisition, was impaired in the mutant (Supplementary Fig. S3)^19,46,48^. However, this characteristic may be irrelevant when *Lp* is exposed to water, as genes related to pigmentation and iron acquisition (*lpg2278*/*hpd*/*lly*, *feoA*, *lpg0232*,  *lpg0124*, *lpg0746*, *lpg0467*, *np20*) were repressed by the TCS (Table 1)^71–73^. The absence of *letS* also caused a slightly elongated cellular morphology in water compared to the WT (Fig. 2).

Importantly, an intact LetA/S system is required for culturability of *Lp* in water (Fig. 1). We corroborate previous studies showing that the LetA/S cascade exerts its regulatory activity via the two small RNAs, RsmY/Z during PE phase in broth (Fig. 7B)^46^. Defects previously associated with the LetA/S system occur under the starvation conditions found during the PE and transmissive phases^19,50,69^. Our data show that LetA/S is also activated in water, leading to RsmY and RsmZ expression (Fig. 7B). Interestingly, the transcriptomic changes initiated by the LetA/S cascade occur and are needed rapidly upon water exposure (Fig. 1).

LetS is a tripartite sensor kinase whose ortholog, BvgS, in *Bordetella* is known to respond to multiple environmental stimuli, activating specific regulons^22,74^. Accordingly, differences between the LetS regulon of *Lp* in water and that in the PE phase were expected^46,50,75^. While both are characterized by the absence of nutrients, the latter accumulates waste products likely causing additional stress. Indeed, *Lp* can survive for several weeks in Fraquil, but dies after only a few days in broth^11^. Over time, WT *Lp* exposed to water progressively shuts down transcription relative to exponential growth^8^. In stark contrast, an overwhelming majority of differentially expressed genes are up-regulated (97%) in the absence of *letS* (Fig. 4). In PE phase, approximately equal numbers of genes are positively and negatively influenced by the LetA/S cascade^46^. In water, LetS almost exclusively represses genes encoding functions that are unnecessary for survival, such as translation and metabolism of amino acids, lipids and carbohydrates (Fig. 6).

Over-expression of replicative phase genes in the absence of LetS is likely due to its downstream effect on CsrA. CsrA affects mRNA expression by: 1) blocking the ribosome binding site, 2) increasing degradation by recruiting RNase E, or 3) enhancing the stability and, thereby, expression of the target mRNA^76^. In ∆*letS*, binding of CsrA to target mRNAs is not relieved by RsmY/Z, which will favor the continued expression of replicative genes, while repressing transmissive genes^48^. A recent study revealed that CsrA directly regulates 479 transcripts involved in amino acid metabolism, carbon metabolism, virulence, flagella expression and iron acquisition^77^. For example, the binding of CsrA to the *gap* mRNA increases transcription by preventing rho-dependent termination^77^. As a result, *gap* expression increased in the absence of LetS in water (Supplementary Table S1). Moreover, two out of three Fis transcriptional regulators (Lpg0542/Fis1 and Lpg1743/Fis2), that are stabilized by CsrA^77^, are repressed by LetS in water. Strikingly, 30 ribosomal genes were highly up-regulated in the absence of *letS* (Supplementary Table S1). In contrast, only five ribosomal genes are reported to bind CsrA directly^77^, suggesting indirect regulation by CsrA. Another notable regulator repressed by LetS in water is the housekeeping sigma factor, RpoD. Increasing the amount of RpoD in the *letS* mutant strain could interfere with the binding of other sigma factors to the core RNA polymerase, a mechanism termed sigma factor competition^78^. Regulation of RpoD by LetA/S or by CsrA has not been reported in *Lp*^46,48,77^. As such, it is unclear whether the effect of CsrA on *rpoD* in water is direct or indirect. The general down-regulation of gene expression upon LetS activation in water is likely mediated by relieving both the direct and indirect effects of CsrA binding to target mRNAs.

The most noticeable difference between the transcriptome of the *letS* mutant in PE phase and in water is the marked absence of gene up-regulation. Only 16 genes were significantly (*p* < 0.05) induced by LetS in water, including RsmY/Z that are directly regulated by LetA (Table 1). In contrast, over 300 genes were significantly up-regulated in the PE phase when *letA* or *letS* were absent^46^. This is presumably because water is a less stressful condition than PE phase, during which metabolic by-products accumulate. As such, virulence regulators (RpoS, LetE and LqsR) were not affected by LetS in water (Supplementary Table S1). While LetA/S controls flagella genes in broth cultures^19,23,46,69,79^, we report that only one flagella-associated gene was differentially expressed in ∆*letS* in water (Supplementary Table S1). At 25 °C, flagella genes are maximally induced at 6 hours after exposure to water^8^. At 42 °C, it is possible that their induction was not captured at the 2-hour time point. Furthermore, flagellar gene expression is tightly regulated in *Lp*, both cooperatively and independently by several high-profile regulatory entities including RpoS, RpoN, FleQ, LetS and LqsR^46,79–81^.

PE phase heat shock (HS) resistance is presumed to be conferred by genes under LetS control^48,50^. Accordingly, ∆*letS* was also heat sensitive relative to the WT after water exposure (Fig. 3); however, similar to flagella gene expression, HS genes were unaffected by LetS at 42 °C. The HS response is initiated within the first few minutes of exposure to this stress, whose transcriptomic effects subside quickly thereafter^82,83^. It is, therefore, possible that the HS-related transcriptomic changes were not detectable by the microarray analysis, if, indeed, they were activated at 42 °C. Alternatively, the general sensitivity of the *letA/S* mutants to various stresses, including heat shock, (Fig. 3)^48,50^ may be a result of cell structure and therefore, a by-product of the cell’s inability to adapt to the respective environments.

Upon sensing starvation, bacteria deploy the stringent response (SR) network governed by the cellular alarmone ppGpp, a key signal in growth phase differentiation^60,84^. SR is characterized by a rapid downshift in the synthesis of stable RNAs like rRNAs and tRNAs^85^. For the most part, ɤ-proteobacteria synthesize ppGpp using RelA, a synthase, and SpoT, a dual-acting hydrolase with weak synthase activity^60,85^. ppGpp positively affects cellular levels of RpoS, an alternative sigma factor^60,86^. Mutation of RpoS leads to the over-expression of replicative genes in water^14^, similar to the *letS* mutant (Fig. 5). Given these transcriptomic similarities, the LetA/S system was proposed to be integrated into the SR during water exposure, which suggests that the same activating signal initiates the LetS cascade.

Northern blotting was used to probe for the direct downstream targets of LetA/S, RsmY and RsmZ (Fig. 7B). Both sRNAs were LetS-dependent and highly expressed upon water exposure (Fig. 7B). The ppGpp° strain (∆*relA∆spoT*) that is unable to survive in water^14^ was used to determine the effect of the alarmone on RsmY/Z production. We show that the ppGpp° strain did not change RsmY/Z expression considerably (Fig. 7B). Therefore, the transcriptomic changes initiated by the SR seem largely independent of those mediated by LetS, and the survival defect of ppGpp° is not due to an impaired LetS response and vice-versa. We also confirm that RsmY/Z expression is RpoS-dependent in water (Fig. 7C)^65^. ppGpp may contribute to RpoS-mediated RNAP binding to *letS* or *rsmY/Z*, but its effect is likely minimal (Fig. 7B). Notwithstanding its effect on transcription, the LetA/S-RsmY/Z-CsrA cascade and the SR seem to be parallel responses contributing to survival under nutrient-deprived conditions. The data presented here suggests the following model. ppGpp and the LetA/S cascade regulate similar regulons in parallel and possibly in different time frames, each promoting adaptation to and survival in water independently. A ppGpp° mutant has a survival defect that is apparent earlier than that of the *letS* mutant in water^14^, suggesting that the SR is more important than LetS and that its effect is required immediately upon exposure to water. RpoS which is positively affected by ppGpp, positively influences the *letS* transcript and thereby, levels of RsmY/Z (Fig. 7C). However, whether RpoS increases the level of *letS* in response to ppGpp in water, or whether basal RpoS levels maintain constant *letS* expression within the cell is not yet know.

It is likely that LetS responds to a variety of stresses fine-tuning the transcriptomic response to the challenge at hand^23^. In broth, LetA/S was required to increase flagella expression in response to nicotinic acid, and the expression of several virulence traits in response to free fatty acids^75,87^. It is unclear whether these are direct signals and whether they also represent activation signals in water. Recently, the *Lp* quorum sensing molecule, *Legionella* autoinducer-1 (LAI-1) was shown to increase RsmY/Z levels in broth^88^; however, it is unlikely to be a viable signal for the activation of LetS in water, because of the bacterium’s low metabolic activity, lowering LAI-1 production under this condition. As such, the environmental signal initiating the LetS/A cascade in water is yet to be determined. This study does not exclude the possibility that ppGpp is one of multiple stimuli sensed by LetS in water; its weak contribution to LetS activation may be masked by other, stronger signals.

In conclusion, we report that the LetA/S-RsmY/Z-CsrA regulatory cascade is essential for the culturability of *Lp* in water. In contrast to the transcriptome in broth cultures, LetS almost exclusively acts to repress genes related to growth; with RsmY/Z being the prominent exceptions. While there is overlap between the regulons and crosstalk between members of the SR and the LetA/S TCS, activation of the two systems seems to be independent of each other in water.

## Experimental Procedures

### Bacterial strains and media

KS79 is a ∆*comR* mutant of the JR32 strain rendering it constitutively competent. JR32 is a salt-sensitive, streptomycin-resistant, restriction negative mutant of *Lp* strain Philadelphia 1^89^. The increased competence of KS79 renders allelic exchange mutations through natural transformation possible. It was, therefore, used as the wild-type (WT) strain in this study. A complete list of strains used in this study can be found in Table 2. Bacterial strains stored at −80 °C in 10% glycerol were grown on CYE (ACES-buffered charcoal yeast extract) agar supplemented with 0.1 mg ml^−1^ α-ketoglutarate, 0.25 mg ml^−1^ L-cysteine and 0.4 mg ml^−1^ ferric pyrophosphate^90^. AYE broth (CYE without agar and charcoal) was used as the liquid medium^90^. When necessary, media were supplemented with 5 μg ml^−1^ chloramphenicol, 2.5 μg ml^−1^ kanamycin and/or 0.1 mM IPTG.

**Table 2:** Strains used in this study.

Strain Name	Relevant Genotype^a^	Source or Reference
*Legionella pneumophila*		
JR32	Philadelphia-1; Sm^r^; r^-^ m^+^	^89^
KS79 (WT)	JR32 ∆*comR*	^96^
∆*letS* (GAH338)	KS79 *letS*::*aptII*, Kn^r^	^65^
*∆letS* + p*letS* (SPF39)	*∆letS* + pMMB207c *Ptac-letS*; Cm^r^	^65^
∆*rsmYZ* (SPF41)	KS79 *rsmY::aptII, rsmZ::aacC1*; Kn^r^, Gm^r^	This study
*∆rsmYZ* + p*rsmY* (SPF291)	*∆rsmYZ* + pXDC39-p*rsmY*; Kn^r^, Gm^r^, Cm^r^	This study
∆*rpoS*	JR32 *rpoS*::Tn903dGent; Gm^r^	^96^
*∆rpoS* + p*rpoS*	**∆***rpoS* + pMMB207c *Ptac-rpoS*; Cm^r^	^14^
∆*relA*∆*spoT* (ppGpp°)	KS79 ∆*relA*::*aacC1 spot::aptII*; Gm^r^, Km^r^	^14^
Plasmid Name		
pBBR1MCS-5	pBBR1MCS Gm^r^	^97^
pSF6	DH5α, pGEMT-easy-*rrnb*	^98^
pMMB207c	RSF1010 derivative, IncQ, lacI^q^ Cm^r^ P*tac oriT* ∆*mobA*	^99^
pXDC39	pMMB207c ∆*Ptac*, ∆*lacI*, Cm^r^	Xavier Charpentier
prsmY	pXDC39-*rsmY*, Cm^r^	This study

^a^Sm^r^, streptomycin resistance; Cm^r^, chloramphenical resistance; Gm^r^, gentamicin resistance; Km^r^, kanamycin resistance.

### Deletion of *rsmY* and *rsmZ*

Construction of the ∆*rsmYZ* strain was performed by allelic exchange as described before^65^, by replacing, first, *rsmY* with a kanamycin resistance cassette and, then, *rsmZ* with a gentamicin resistance cassette. Two 1-kb fragments corresponding to the upstream and downstream of *rsmY* were amplified using primers rsmY-BF/rsmY-BR and rsmY-EF/rsmY-ER, respectively. A kanamycin cassette was amplified from pSF6 using primers rsmY-BRKN/rsmY-EFKN. PCR fragments were purified on gel using a gel extraction kit (Qiagen). The three fragments were ligated together by PCR using primers rsmY-BF/RsmY-ER. The resulting 3 kb fragment was purified on gel, as described above, and introduced into KS79 by natural transformation^91^. The recombinants were selected for kanamycin resistance and the allelic exchange confirmed by PCR. Deletion of *rsmZ* was performed similarly by using primers rsmZ-BF/rsmZ-BR and rsmZ-EF/rsmZ-ER to amplify the upstream and downstream fragments, and rsmZ-BRGT and rsmZ-EFGT to amplify a gentamycin cassette from pBBR1MCS-5. The resulting 3 kb fragment was introduced into the ∆*rsmY* strain, recombinants were selected for kanamycin and gentamicin resistance and the deletion of *rsmZ* was confirmed by PCR. The resulting strain was named SPF41. Northern blot was used to confirm absence of expression of both sRNA. All PCR amplifications were carried with Phusion polymerase (NEB) according to the manufacturer’s protocol. Primer sequences used in this study are found in Supplementary Table S2.

### Cloning of *rsmY*

The *rsmY* gene was amplified with its own promoter from the KS79 wild-type strain using primers rsmY-F and rsmY-R (Supplementary Table S2). The pXDC39 vector and the amplicon were then digested with BamHI and HindIII (New England Biolabs) according to the manufacturer’s protocol, and purified using a MinElute purification kit (Qiagen). The digested vector and insert were ligated overnight using T4 DNA Ligase (New England Biolabs) at 16 °C. The recombinant plasmid was then transformed into *E. coli* DH5α (pSF86). The transformed population was incubated at 37 °C shaking for 90 minutes before plating on 25ug ml^−1^ chloramphenicol plates. Colonies that grew on antibiotic plates were patched and tested by PCR for insertion of the *rsmY* gene into the vector. The recombinant plasmid was extracted using a plasmid extraction kit (Qiagen) and introduced into the ∆*rsmYZ* mutant to produce the ∆*rsmYZ* + p*rsmY* strain (SPF291).

### Survival in water

Survival in water was tested in the artificial freshwater medium, Fraquil, as described previously^8,51^. Briefly, *Lp* strains cultured on CYE agar at 37 °C for 3 days were suspended in Fraquil at an OD_600nm_ of 0.1 and washed three times with Fraquil. One millilitre of this bacterial suspension was mixed with 4 ml of fresh Fraquil in a 25 cm^2^ cell culture flasks (Sarstedt) and incubated at 4 °C, 25 °C or 42 °C. To test survival in water at high cell densities, strains were suspended in Fraquil at an OD_600nm_ of 1 and washed three times with Fraquil. Five millilitres of this bacterial suspension was placed in a 25 cm^2^ cell culture flasks (Sarstedt) and incubated at 42 °C. Survival of the strains in water was monitored using CFU counts.

### Microscopic analysis

*Lp* strains were grown on CYE agar for 3 days at 37 °C. AYE broth was inoculated with the respective strains and grown to exponential phase (OD_600_ 0.4–0.7) or post-exponential phase (OD_600_ > 3) at 37 °C shaking (200 rpm). To test the effect of water, strains were suspended in Fraquil at an OD_600_ of 1 and incubated at 42 °C for 24 hours. 20 μl of each sample was placed on a clean microscope slide, covered with a cover slip and observed under 1000X magnification under oil immersion using digital phase contrast microscopy (Nikon Eclipse 80i). For each strain, 10 images of random microscopic fields were captured using the NIS Element Software (Nikon Instruments, Inc.). ImageJ software^92^ was used for quantitative analysis of cell length. 10 cells from 10 different fields of view were randomly chosen and analyzed per strain per treatment (n = 100). Multiplying cells (presence of a septum) were excluded and only individual, non-filamentous cells were used for analysis.

### Pigment production

*Lp* strains grown on CYE for 3 days at 37 °C were inoculated into AYE broth. Strains were grown to late post-exponential phase at 37 °C shaking at 200 rpm. 10 ml of each strain was pelleted at 4500 rpm for 10 minutes. The supernatant was then removed and filtered using 0.2μm pore sized syringe filter. The optical density of the supernatant was measured at 550 nm.

### Heat shock

*Lp* strains cultured on CYE agar at 37 °C for 3 days were suspended in Fraquil at an OD_600nm_ of 0.1, as described above for the survival assay. One milliliter aliquots of each strain were transferred to 13 ml tubes (Sarstedt) and were allowed to acclimate to the water environment for 2 hours at room temperature. At the end of the incubation period, tubes were submerged in a 55 °C water bath. At each time point tested, three biological replicates from each strain were removed from the water bath and the CFU counts enumerated on CYE agar.

### DNA microarray

The WT, ∆*letS*, and the complemented strain induced (ON) or not (OFF) with IPTG were grown on CYE agar for 3 days at 37 °C. Each culture was suspended in Fraquil in triplicate at an OD_600_ of 1. 20 ml of each strain was placed in 75 cm^2^ cell culture flasks and incubated at 42 °C for 2 hours. After incubation, 10 ml aliquots were pelleted for 5 minutes at 5000 g. After centrifugation, the supernatant was removed, and the cell pellets were re-suspended in 1 ml of TRIzol reagent (Invitrogen). Three biological replicates of each strain were used for the transcriptomic analysis. The three remaining replicates of each strain were preserved at −20 °C for further experimentation. RNA extractions were done according to the manufacturer’s protocol. To remove DNA contamination, extracted RNA was subsequently treated with Turbo DNase (Ambion) as per the manufacturer’s protocol. The purity and concentration of RNA were determined by UV spectrophotometry. Fifteen micrograms of RNA was labeled with aminoallyl-dUTP (Sigma) during reverse transcription (ProtoScript II, New England Biolabs) using random hexamers (Life Technologies) as previously described^8,93^. Genomic DNA was used as a reference channel and was labeled by random priming using Klenow fragments (New England Biolabs), aminoallyl-dUTP and random primers as described previously^93^. The cDNA and gDNA were subsequently coupled to the succinimidyl ester fluorescent dye (Life Technologies) AlexaFluor 647 or AlexaFluor 546, respectively, following the manufacturer’s protocols. The microarray design (GPL19458) and the protocol for hybridization, data acquisition and data analysis have been published previously^8^. Statistical analyses were performed using an unpaired one-tailed Student’s *t*-test. Genes were considered differentially expressed if they demonstrated a ratio-to-control value of ± 2-fold with a *P* < 0.05.

### qPCR validation

One replicate of the WT, ∆*letS* and the complemented strain induced (ON) or not (OFF) with IPTG exposed to water as described above was used to validate the microarray results using qPCR. RNA was extracted as described above. 1 μg of total RNA was transcribed to cDNA (Supercript II, Life Technologies) using random hexamers. qPCR was performed as described previously using the primers described in Supplementary Table S2. Ct values were normalized to the 16 S rRNA.

### Northern Blotting

*Lp* strains grown on CYE agar for 3 days at 37 °C were used to inoculate AYE broth. All strains were grown to exponential phase (OD_600_ 0.4–0.7) at 37 °C shaking (200 rpm). For each strain, 10 ml of exponential phase bacterial culture was centrifuged for 10 minutes at 4500 rpm, the supernatant removed and the pellet re-suspended in 10 ml of Fraquil. Water-exposed bacteria were incubated at 42 °C for 2 hours, after which cells were pelleted and suspended in 1 ml of TRIzol reagent (Invitrogen). RNA was extracted according to the manufacturer’s protocol. RNA from the WT was also extracted from 10 ml of exponential phase culture and 5 ml of post-exponential phase culture (OD_600_ > 3). 1 ug of RNA was loaded and migrated on a 6% Tris-borate-EDTA-urea polyacrylamide gel (Ambion) at 180 mV. The RNA was transferred onto a positively charged nylon membrane (Thermo Scientific) using a semidry gel blotting system (BioRad) for 20 minutes at 200 mA. The membranes were pre-hybridized in ULTRAhyb-Oligo Hybridization Buffer (Ambion) for 1 hour at 37 °C before hybridization with 5′ biotinylated RsmY and RsmZ probes (Integrated DNA Technologies). Hybridization was performed overnight in a rotating chamber at 37 °C. Blots were washed twice with 2X SSC (0.15 M NaCl and 0.015 M sodium citrate) and 0.5% SDS for 30 minutes. The biotinylated probed were detected using the Chemiluminescent Nucleic Acid Detection Module (Thermo Scientific) as per the manufacturer’s instructions. The exposure time used for image acquisition was 1 second.

### Bioinformatic analyses

NCBI Conserved Domain Database (CDD https://www.ncbi.nlm.nih.gov/cdd/) was used to search for conserved protein domains that may be implicated in signal sensing within LetS. The Accession number YP_095929.1 representing Lpg1912 of *Legionella pneumophila* Philadelphia-1 strain was queried. The TMHMM server v. 2.0 (http://www.cbs.dtu.dk/services/TMHMM/) and TOPCONS (http://topcons.net/) were used to predict the transmembrane helices on the N-terminal of LetS^94,95^.

### Availability of materials and data

Microarray data generated during the current study are available from Gene Expression Omnibus (GSE98743).

## Electronic supplementary material

Supplementary Figures and Table S2

Dataset 1
