# Supplementary material for: The LetA/S two-component system regulates transcriptomic changes that are essential for the culturability of Legionella pneumophila in water
Source: Sci Rep. 2018 Apr 30;8:6764. doi: 10.1038/s41598-018-24263-9 (PMC5928044; doi:10.1038/s41598-018-24263-9)
Supplement: Supplementary file 1 — Supplementary Figures and Table S2 [file 41598_2018_24263_MOESM1_ESM.pdf]

The LetA/S two-component system regulates transcriptomic changes that are essential for the culturability of *Legionella pneumophila* in water

Nilmini Mendis[a], Peter McBride[a], Joseph Saoud[a], Thangadurai Mani[a], and Sebastien P. Faucher[a,]*

[a]Department of Natural Resource Sciences, McGill University, Sainte-Anne-de-Bellevue, Quebec, Canada.

*Corresponding author, e-mail sebastien.faucher2@mcgill.ca.

Supplementary Figures

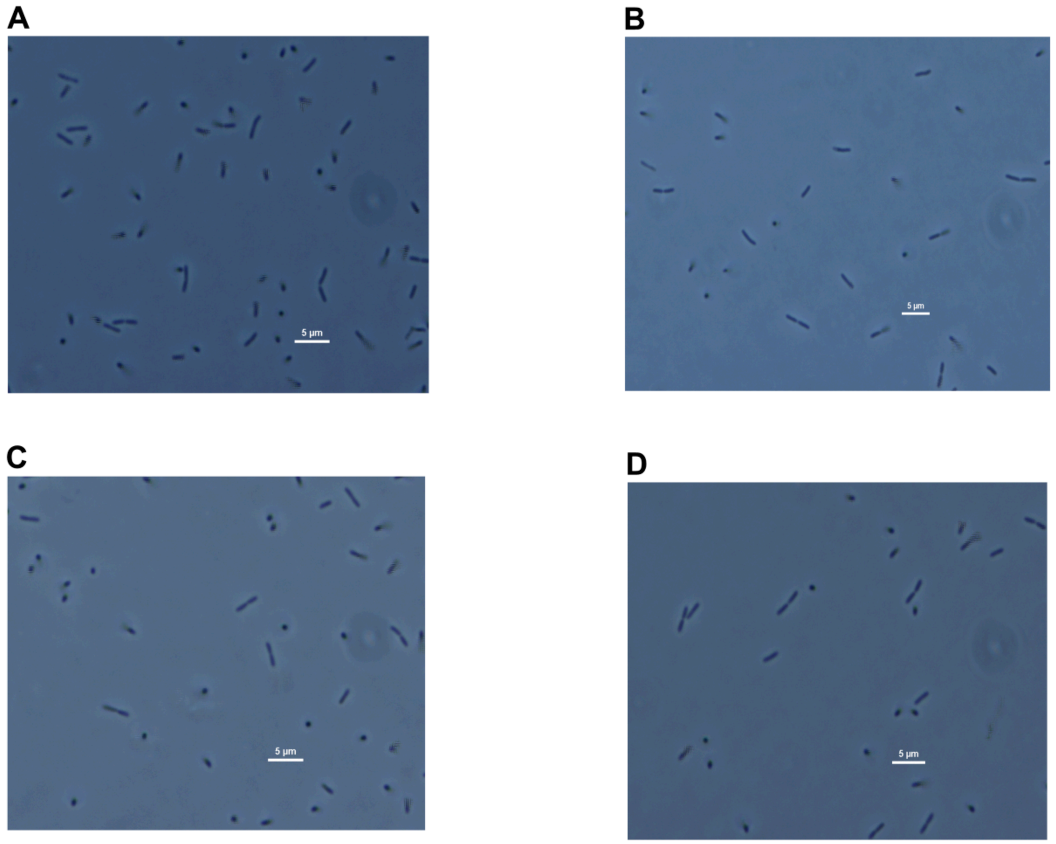

Figure S1: Microscopic images of exponential phase cells. A representative image of the WT (A), Δ*letS* (B), induced (ON) or uninduced (OFF) Δ*letS* + p*letS* grown to exponential phase. The induced complementing strain (ON) was grown with 0.1mM IPTG. Phase contrast microscopy was used to visualize morphological changes at 1000X magnification under oil immersion. The scale bar is equivalent to 5µm.

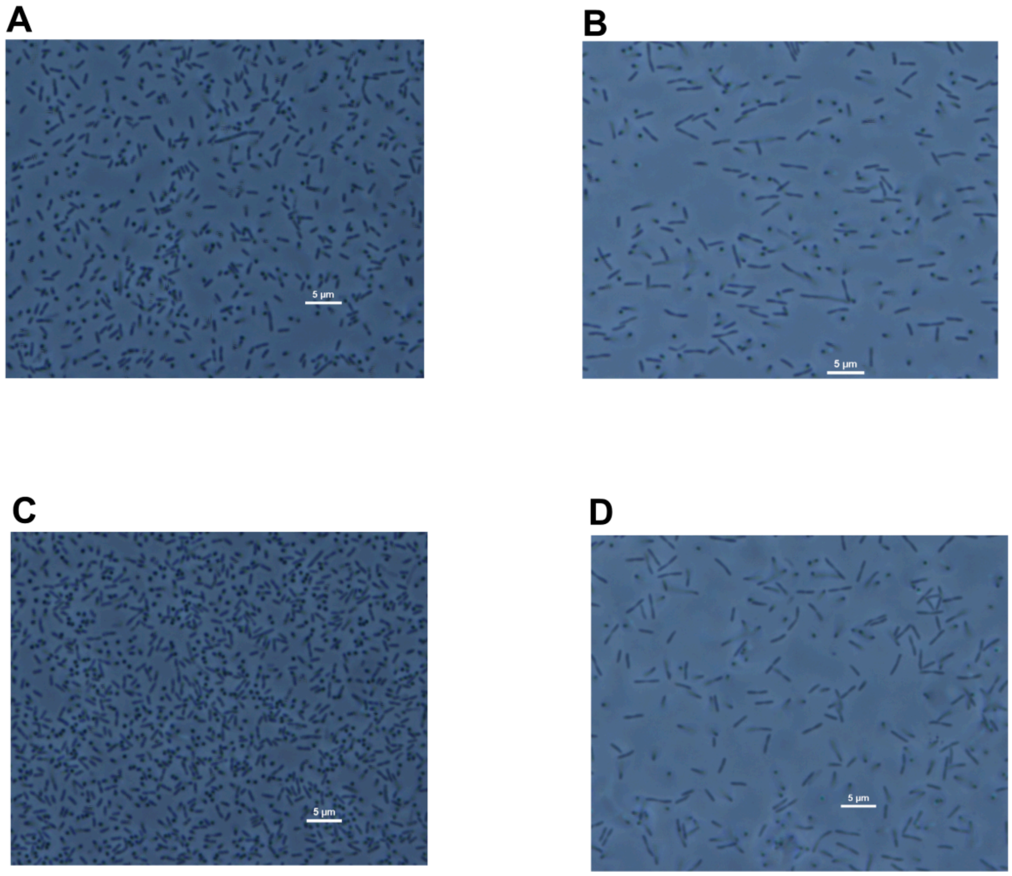

Figure S2: Microscopic images of post-exponential phase cells. A representative image of the WT (A), Δ*letS* (B), induced (ON) or uninduced (OFF) Δ*letS* + p*letS* grown to exponential phase. The induced complementing strain (ON) was grown with 0.1mM IPTG. Phase contrast microscopy was used to visualize morphological changes at 1000X magnification under oil immersion. The scale bar is equivalent to 5µm.

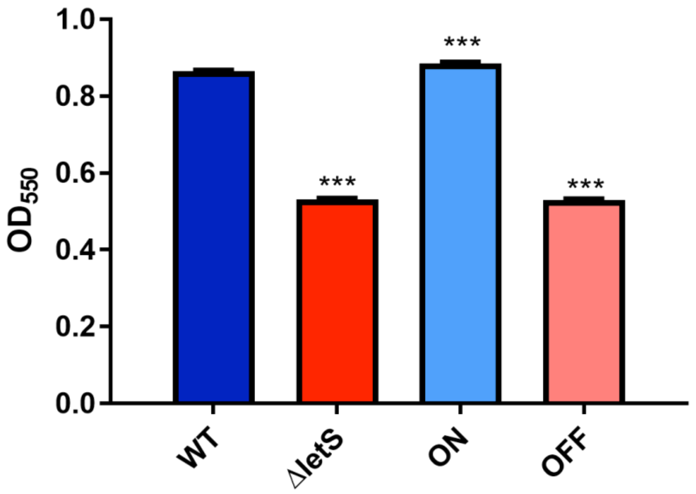

Figure S3: Deletion of *letS* affects pigment production. The WT, Δ*letS*, induced (ON) or uninduced (OFF) Δ*letS* + p*letS* grown to late post-exponential phase in AYE broth at 37°C. The optical density (OD_{550}) of 1ml of cell-free supernatant was measured in triplicate. An unpaired, one-tailed Student's t-test was used to assess statistical significance *versus* WT. *** $P < 0.0005$.

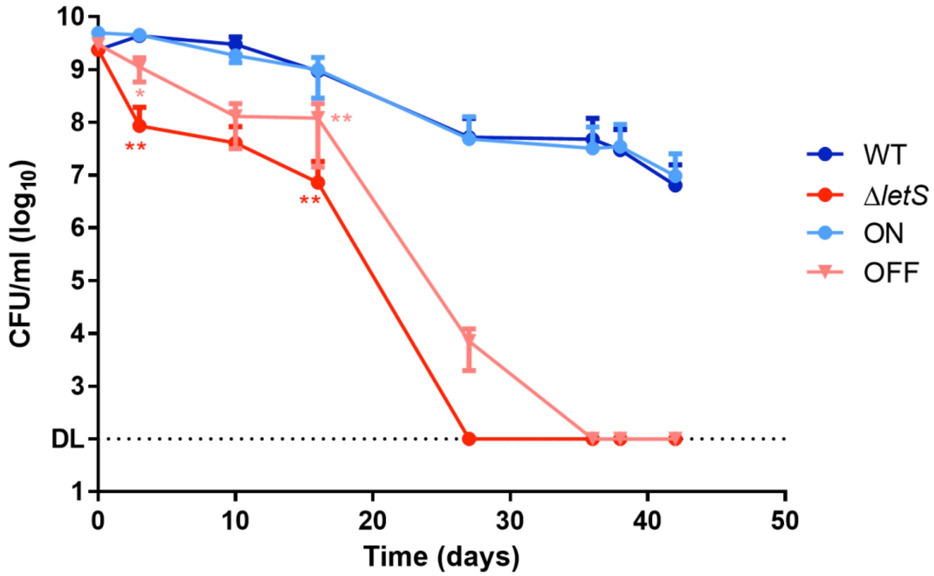

Figure S4: The survival of *Lp* in water at an OD_{600} of 1 at 42°C. The WT, Δ*letS* and the induced (ON) or uninduced (OFF) Δ*letS* + p*letS* were exposed to 42°C at OD_{600} of 1. ON was induced using 0.1 mM IPTG on agar prior to water exposure and with 0.1 mM IPTG during water exposure. DL, detection limit. An unpaired, one-tailed Student's t-test was used to assess statistical significance *versus* the WT. * $P < 0.05$; ** $P < 0.005$; *** $P < 0.0005$.

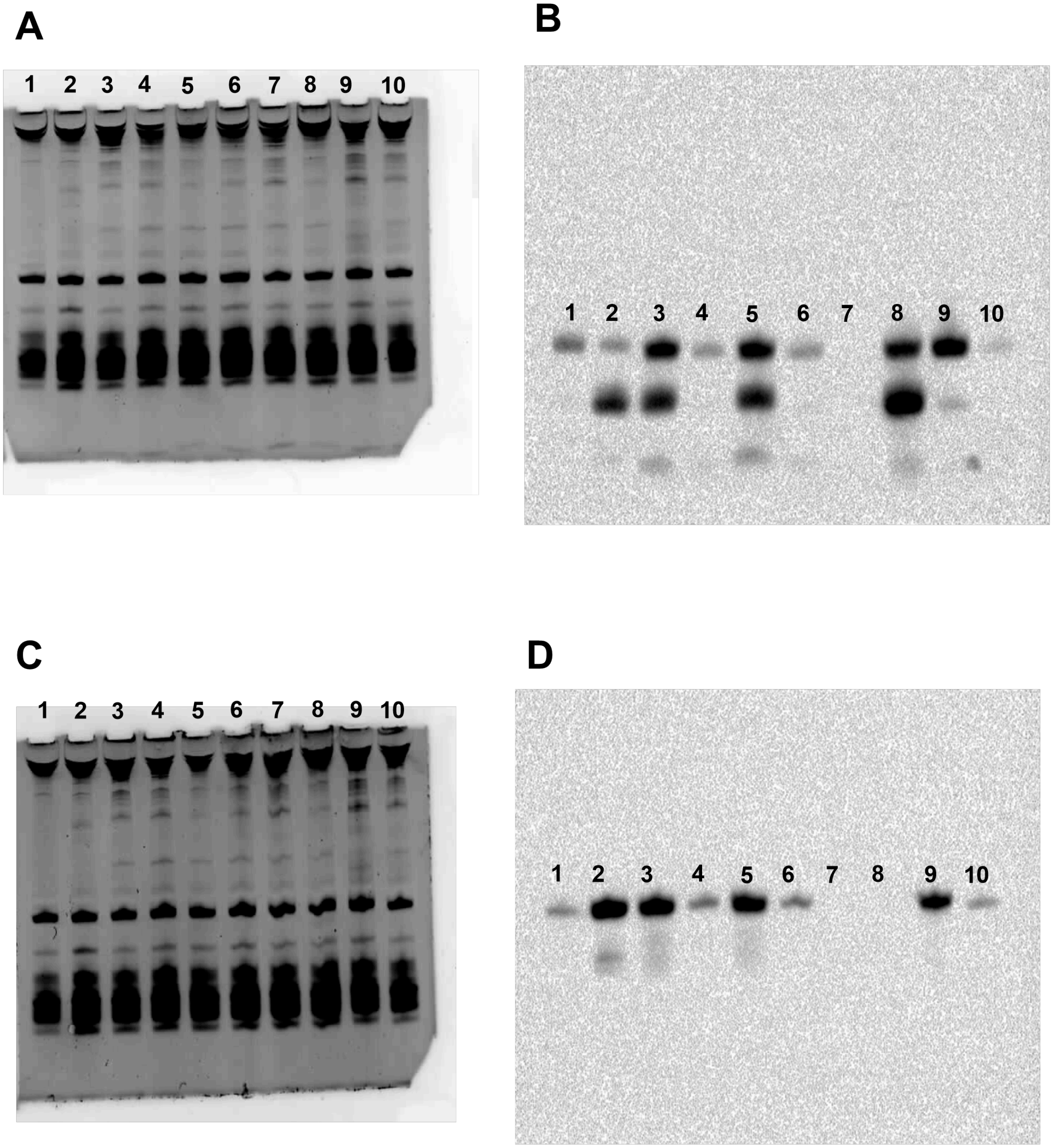

Figure S5: Complete gel/blot images of ethidium bromide staining and chemiluminescent detection of RsmY and RsmZ. A) and C) full RNA profiles revealed by ethidium bromide staining of 6% Tris-borate-EDTA-urea polyacrylamide gels. A and C were transferred to nylon membranes to probe for RsmY (B) and RsmZ (D) respectively. 1 = WT in E phase, 2 = WT in PE phase. Lanes 3-10 are strains exposed to water for 2 hours at 42°C. 3 = WT, 4 = Δ*letS*, 5 = ON, 6 = OFF, 7 = Δ*rsmYZ*, 8 = Δ*rsmYZ* + p*rsmY*, 9 = Δ*relAspoT* (ppGpp^{0}), 10 = Δ*rpoS*.

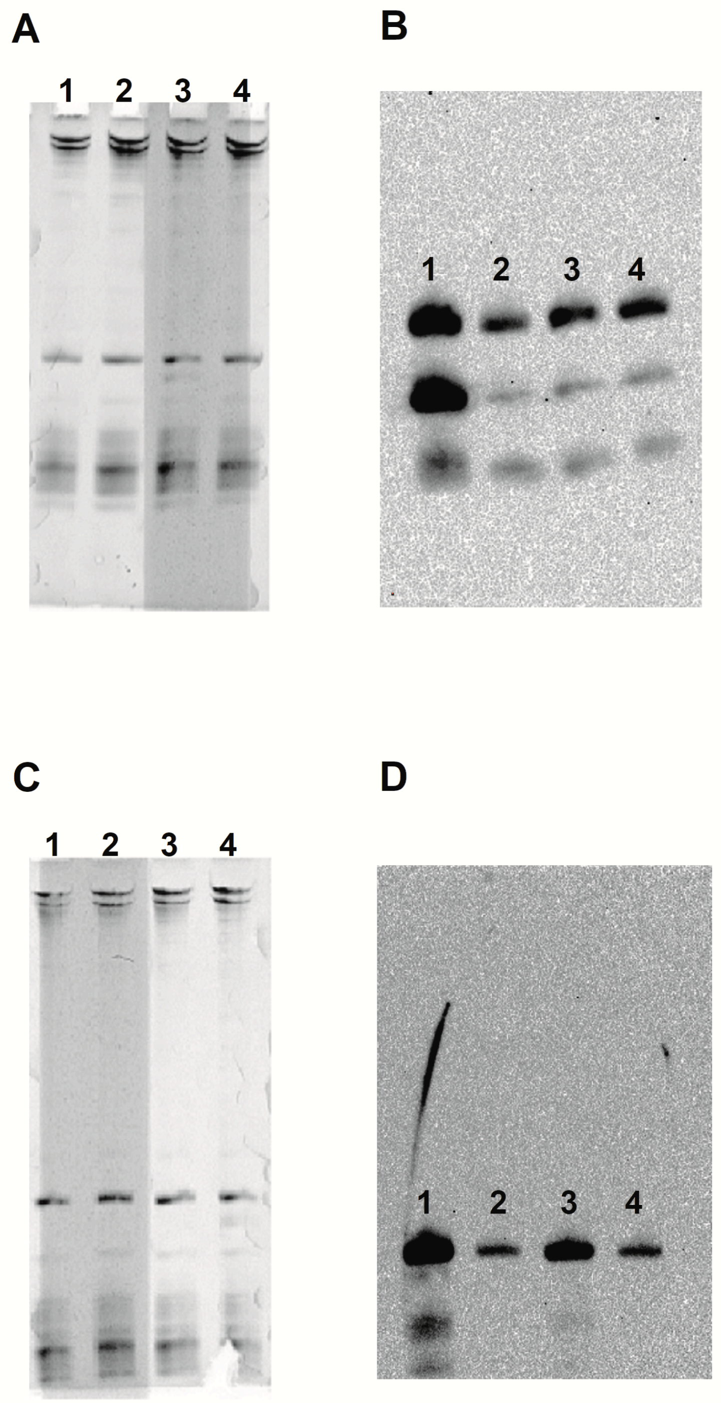

Figure S6: Complete gel/blot images of ethidium bromide staining and chemiluminescent detection of RsmY and RsmZ. A) and C) full RNA profiles revealed by ethidium bromide staining of 6% Tris-borate-EDTA-urea polyacrylamide gels. A and C were transferred to nylon membranes to probe for RsmY (B) and RsmZ (D) respectively. Strains were exposed to water for 2 hours at 42°C. 1 = JR32 (WT), 2 = Δ*rpoS*, 3 = Δ*rpoS* + p*rpoS* induced with IPTG, 4 = Δ*rpoS* + p*rpoS* uninduced.

Table S2: Primers and oligonucleotide probes used in this study

Primers	Sequence 5' – 3'	Source or Reference
rsmY-F	CGGGATCCGCTCCTGGAAAGGTGTTATGC	This study
rsmY-R	CCCAAGCTTAAAGAGGTATACTGGTAAATTG G	This study
rsmZ-F	GGATATGAGTCGTGCAAATGG	This study
rsmZ-R	TTCGCAGTCATCCGTATAAGA	This study
psRNA_lpg2153-F	TAATGAATAGTACAAAGCTGTGGCA	This study
psRNA_lpg2153-R	AAGCAGCAGCTTGATGAGAAA	This study
lpg0879-F	GCGATCGCTTCTCTGTCTATT	This study
lpg0879-R	CATGGTTGCTAACATCGTTCTATC	This study
tig-F	TGGTTCTGGTTCGATGATTCC	This study
tig-R	TCCTTCCCAGCCAAATCTTTAT	This study
mreB-F	GTCTGTGTTCCTTGTGGTTCTA	This study
mreB-R	CAGCCATAGGCTCCTCAATAAG	This study
efp-F	CTGATGTGGCTGACGTAGAAA	This study
efp-R	TTGTGCTGCATCGGCTAATA	This study
rsmY-NB	biotin-GCAGCGAAGTACATCCTTTGTACTG<br>GTCCCTTAGTTGACTTCCTGTCAGACATATCC	(Hovel-Miner *et al.*, 2009)
rsmZ-NB	biotin-CGCAGTCATCCGTATAAGAACTT<br>GCGTTCTTATTGTCATCCTGACAAATC	(Hovel-Miner *et al.*, 2009)
rsmY-BF	TTGATTACTGCATTAGGCTGTGG	This study
rsmY-BR	GCTCTAGACAAACATCACTCCAGTTCAACAA TAC	This study
rsmY-BRKN	CAATGTAACCCGGCCAAGCTCAAACATCACT CCAGTTCAACAATAC	This study
rsmY-EFKN	GCTCGATGAGTTTTTCTAAGGATCCTTAAGA	This study

	AGTGGCATTGTCTTCGTC	
rsmY-EF	GCTCTAGATTAAGAAGTGGCATTGTCTTCGT C	This study
rsmY-ER	CCTGCTGAATGGTATCCTCATG	This study
rsmZ-BF	GGCATATTATATGACGACAATCCTG	This study
rsmZ-BR	GCTCTAGAGGGCTGAGTCCCTGGCTTAC	This study
rsmZ-BRGT	GCAAGGCGACAAGGTGCTGATGGGCTGAGTC CCTGGCTTAC	This study
rsmZ-EFGT	CGTTCAAGCCGAGATCGGCTTCAGCGCAAGG TGTTAAATCACAC	This study
rsmZ-EF	GCTCTAGAAGCGCAAGGTGTTAAATCACAC	This study
rsmZ-ER	AGTGGATTAGATGCAGAACCAGAAG	This study
